# Me too! A case study of gendered victimization and feminist development in a Swedish peer support organization for people with experiences of criminalization and substance abuse

**DOI:** 10.3389/fpsyt.2022.937228

**Published:** 2022-08-02

**Authors:** Emy Bäcklin

**Affiliations:** ^1^Department of Criminology, Stockholm University, Stockholm, Sweden; ^2^Department of Social Work and Criminology, University of Gävle, Gävle, Sweden

**Keywords:** feminism, #MeToo, peer support, reintegration, substance abuse

## Abstract

Even if peer support is commonly defined as horizontal in contrast to the more hierarchical relationship between client and professional, peer support is not free from power dynamics. This article considers feminist organizing in the context of peer support for people with experiences of criminalization and substance abuse and addresses questions of (un)equal peer support, sexual victimization, (re)integration, and organizational change in the #MeToo era. Drawing on qualitative interviews with support organization representatives and discussion material from a study circle and a men’s group, this article analyses one organization’s framing of, and responses to, allegations of sexual victimization of female members, and their ongoing work toward increased equality. The study shows that a number of measures have been taken in the organization in order to give voice to women whose lives are affected by crime, imprisonment, violence, and drug abuse. Interview participants put strong emphasis on the need to counteract what is described as a “macho culture” embedded in the peer support organization (PESO), which is seen as repeating structures of masculinity and power from the previous criminal lifestyle as well as reproducing specific gendered vulnerabilities. The organization’s patriarchal structure is understood as connected to a culture of silence that has allowed for sexism and marginalization of female members to continue. The women’s lived experiences of trauma within peer support practices and their struggles to redefine the foundations of their organization emphasizes the lived gendered emotionality of peer support, and uncovers how power structures can be challenged by putting the gendered lived experiences of women with a history of criminalization and substance abuse in the center of ex-offender peer support.

## Peer support and desistance: A feminist approach

The idea that individuals who have overcome struggles help others with similar challenges dates back a long time. In the literature helpers are referred to as wounded healers (1–4), professional ex-s (5, 6), peer mentors (7), experts by experience (8), and credible messengers (9). The practice of peer support can be informal (for example among friends) or formal/intentional within existing services where people with lived experiences are trained and employed. It can also take place in self-help-oriented groups or PESOs [see (10)]. Peer support and the possibility among persons with lived experiences of incarceration, criminalization, and substance abuse to act as wounded healers has been identified as a practical implication of desistance theory, which in essence is based on the simple idea that people can, and do, change (11, 12). Wounded healers personify the success stories that desistance theory is built upon (12) and their lived experiences often mean that they are perceived as trustworthy (9, 13, 14). Wounded healing has further been highlighted as an effective strategy for scaling up preventive and restorative approaches tackling substance abuse and crime (15, 16). While restorative justice is a normative theory that suggests that harms should be repaired by the harm-doer by means of “giving back,” desistance theory makes the empirical claim that such generative actions support desistance (11, 17, 18). Nugent and Schinkel (19) argue that desistance, in order to be maintained long-term, needs to be supported in three different spheres: the outside world, the world within, and the world of relations to others. They introduce the concepts of act-desistance (non-offending), identity desistance (internalization of an identity as non-offender), and relational desistance (change recognized by others). These spheres are linked to different forms of pains: pain of isolation when trying to achieve act-desistance and pain of goal failure when struggling to form a new identity or experiencing a lack of relational desistance. The importance of being recognized by others as someone who has changed and to be able to develop a sense of belonging to a community is acknowledged in the practice of peer support, where relational and structural aspects of desistance are central. Envisioning the future of desistance research, Maruna (12) highlights the central role of lived experience and argues that desistance should be reframed as a social movement “as that concept moves from the Ivory Tower to the professional world of probation and prisons, back to the communities where desistance takes place” [p. 11; see also (20)]. Indeed, the expression “nothing about us without us” has been key to various social justice movements (21) and is also evident in the field of convict criminology (22), where lived experiences is moved from margins to center by formerly incarcerated academics (12).^1^ Peer support has been described as a political act that builds empowerment by telling and listening to each other’s stories (23). In the spaces created by activist and peer support initiatives people can use their experiential knowledge to advocate for progressive change (23). Furthermore, peer support and peer groups have been referred to as possible safe spaces^2^ for those stigmatized through criminalization and/or substance abuse (24). Nevertheless, even though studies show that peer support is helpful for the helper (2, 25–27), the potential risks or inefficacy of peer support is less explored (28). In the field of mental health, a systematic review and meta-analysis of randomized controlled trials of peer support for people with severe mental illness by Lloyd-Evans et al. (29) found little evidence of the effectiveness of peer support for this particular target group. Other studies indicate that peer support is often most successful “if both parties have other things in common such as cultural background, religion, age, gender and personal values” [(30), s 4; see also (31, 32)].

Peer support is often thought of as horizontal, in contrast to the supposedly more hierarchical relationship between client and professional (7). The sparse research on peer support and mentoring in the criminal justice context has generally not dealt with power dynamics in the peer mentor setting, and Buck (7) points out that “when peer mentoring is framed as a relationship free of authority, one of the major power dynamics which is veiled is gender” (p. 215). Not only is peer mentoring a gendered practice where regulatory gender dynamics are negotiated (7), but peer mentoring spaces such as PESOs are arguably also sexualized spaces in which women with experiences of criminalization and substance abuse may feel less safe and supported, or even become exposed to further victimization (14). Feminist criminology has long since argued that “[g]ender blindness is not a trivial oversight; it carries social and political significance” [(33), p. 98]. Feminist criminologists have shown the extent to which gender matters in the lives of women with experiences of criminalization and substance abuse, not least in relation to the high exposure to violence and sexual victimization (34). Desistance research centering the lived experience of female offenders has further revealed how lingering consequences of prior victimization from violence may restrict women’s routes out of crime (35, 36). Gender blindness thus risks obscuring the various ways in which structural inequalities affect women’s lived experiences of criminalization and substance abuse, as well as their processes of desistance.

## #MeToo and women with experiences of criminalization and substance abuse

One of the goals of the feminist movement has been to “rewrite the scripts that trigger shame” [(37), p. 42] by challenging stigmatizing perceptions of sexual victimization. As a “shame management process,” Maruna and Pali [(37), p. 42] claim that #MeToo, rather successfully, have turned the tables on victim shaming and blaming, and redirected it onto offenders and the society and institutions that have failed to adequately acknowledge and deal with sexual violence and harassment. A large field of research has developed that sheds light on the global development of the #MeToo-movement (38–51). This emerging field is diverse and includes analyses of #MeToo-appeals (52), how managers and management teams have dealt with intra-organizational accusations of sexual harassment (53), effects of #MeToo on attitudes toward sexual assault (54), on reporting workplace sexual harassment (55), and effects in specific work sectors (56).

#MeToo has been described as a movement “concerning sexual harassment at work” [(57), p. 581]. Nevertheless, the limits of the movement are not clearly defined and there has been discussions as to whether it should include non workplace-related victimization (58). Moreover, certain stigmatized groups, such as sex workers and victims of prison rape have expressed doubts about their experiences are welcomed in the #MeToo narrative (58). Questions of the scope of the #MeToo-movement is relevant in relation to women with experiences of substance abuse and criminalization, whom as a group are often marginalized from the mainstream labor market, highly exposed to sexualized violence and abuse (34, 35, 59–62) and suffer from poorer health and living conditions in general (63–66). Moreover, the consequences of the failure to live up to idealized notions of the victim has been discussed in relation to drug using women subjected to partner violence. These women’s drug use is often seen as causing the violence (67). Women who engage in criminal behavior or who use drugs risk being treated as doubly deviant for breaking the law as well as the conventional norms of femininity (36, 68, 69).

In Sweden, the #MeToo-movement had a wide impact. The number of hashtags was, relative to the size of Sweden’s population, highest in the world (45). Between 2017 and 2018, 76 petitions were published in Swedish media (41). Most of the petitions came from different lines of businesses and industries, but several were initiated by groups of (mainly) women not connected to a specific industry sector. Out of the 76 petitions, two were initiated by people with experiences of drug use, sex for compensation and/or criminalization: #withoutsafetynet (#utanskyddsnät) and #notyourwhore (#intedinhora). #Withoutasafetynet’s petition highlighted the fact that women^3^ with experiences of drug use, sex for compensation and/or criminality are rarely treated or viewed as ‘‘fellow humans,’’ seldom included in the imagined sisterhood of the women’s movement, and not sufficiently acknowledged and protected by the welfare state. The initiators of #notyourwhore^4^ defined themselves as joined not by industry, but by their shared vulnerability. Their petition, like #withoutasafetynet’s, made visible the lack of legal rights that characterizes the lives of people with experiences of drug use, sex for compensation and/or criminalization.

## The present study

This article will consider the feminist organizing that has been taking place in KRIS,^5^ a Swedish PESO for people with experiences of criminalization and substance abuse, in the context of #MeToo and in the aftermath of internal accusations of sexual harassment in KRIS. By exploring the organization’s framing of the problem, the responses to victimization of female members, and their ongoing work toward increased equality, the study uses KRIS as a case to address questions of (un)equal peer support and feminist organizing in the context of ex-offender (re)integration.

## Methods

### Context of the study: The KRIS organization

Founded in 1997 by a group of men with a history of incarceration and substance abuse, KRIS is the first and most well-known non-profit PESO for people with experiences of criminalization and substance abuse in Sweden. One of the core activities of the organization is “muckhämtning,” which means that peers pick up prisoners upon release and drive them to a KRIS premises where they have a release-celebration [cf. (11, 70) on re-entry rituals]. Above this, KRIS offers a range of different support services, which differs somewhat between the local associations depending on human and financial resources. At the time of writing this article, KRIS have local associations in eleven Swedish cities^6^ and youth associations (target group 13-25 y/o) in eight^7^. These numbers have fluctuated over the years with several local associations having shut down, and new ones opened.

KRIS frequently comment on criminal policy related issues in Swedish media and participate in different social and political events like Almedalen Week.^8^ Due to KRIS’ high visibility as an organization for liminal groups (71) of people with criminal records and previous addictions, they attract publicity around their projects, activities, and funding. Recently Swedish Television reported that The Swedish Inheritance Fund demands a refund of almost SEK 1MM after finding deficiencies in the financial accounts of KRIS national association (72). This further led to the Swedish Prison and Probation Service denying KRIS state funding for targeted visiting activities and release support in 2021.^9^

An incident that is of relevance in this article is the allegations of sexual harassment of female KRIS members that were directed at the then president of KRIS in 2019 (73). In KRIS magazine *Vägen Ut*, this event and its aftermath are described as something of a turning-point for KRIS as an organization, where a fifth maxim: *Equality*, was added in 2021 [(74), issue 3]. In recent years several projects have started that aim to raise awareness about violence and sexism, work against shame and stigma, and to increase gender equality within the organization.

The current study draws on five semi-structured interviews with seven employed peer mentors working in KRIS. The study also draws on study material from KRIS’ study circle: *The meaning of violence* and discussion questions used in KRIS Stockholm’s weekly men’s group (see Table 1). This study material is used to gain insight on how KRIS implement the equality maxim in their peer support practices.

**TABLE 1:** KRIS projects.

The faces of violence^1^	The project started because of increased isolation of predominately women and children due to the corona pandemic but takes a broader approach in discussing gender-based violence. The project includes a podcast.
The meaning of violence^2^	A study circle for both male and female KRIS members. The study circle is made up of 17 sessions based on Isdal’s book (88) with the same name. The sessions focus on different types of violence such as physical, psychological, material, sexual, economic, and latent violence. The participants read extracts from Isdal’s book before each session, during which they discuss different themes based on between three and six predetermined questions.
Men’s group	A weekly men-only group where the participants discuss questions related to masculinity, sexism, violence, victimization, and family. Offered in the local association KRIS Stockholm.
Women’s groups	Weekly women-only groups where participants are invited to discuss themes including shame and stigma, boundaries, victimization, and violence. Offered in several local KRIS associations.

^1^In Swedish: Våldets ansikten.

^2^In Swedish: Meningen med våld.

In addition to the interviews and study material, I have followed KRIS development through their membership magazine: *Vägen Ut*, which has been distributed quarterly since 2002. Moreover, I have followed KRIS’ social media channels for several years, not least during the period of internal conflicts 2019 surrounding the allegations of sexual harassment against the then president of KRIS (75). I also participated in a seminar on sexism and macho culture held by activist, journalist, and writer Atilla Yoldas, hosted by KRIS Stockholm in February 2021. Above that, I have listened to KRIS’ podcast *The faces of violence* (Våldets ansikten). Even though these sources do not make up explicit empirical material in this study, they formed the rationale for doing the study and influenced the interview questions. Having followed KRIS for many years was also a resource during the interviews in terms of building trust with the interviewees.

### Procedure

The five local KRIS associations that have started one or more gender equality related projects were contacted via their Facebook pages and/or email addresses with an information sheet and consent form for the study. I gave my contact information and asked people that would be willing to be interviewed to contact me. I also contacted specific individuals that I knew were involved in equality projects in KRIS. Through one of them I was referred to several others involved, four of which were subsequently interviewed. After giving informed consent, participants from three local associations were interviewed in person at their local association (n = 2) or over phone (n = 5). In one of the interviews three interviewees participated. This interview differed from the individual interviews, involving discussions and interactions between the participants. Having worked together for several years, they knew each other well and requested to be interviewed together. They had experiences of working in different local KRIS associations and with various assignments in the organization, which suited the purpose of discussing organizational developments.

Conducting qualitative interviews over phone is often considered inferior to face-to-face interviews, although there is no evidence that phone interview data would be of less quality (76). Some studies suggests that phone interviews have the advantage of being more flexible in terms of geography and time scheduling, and that phone interviews can feel less intrusive for the interviewees (76, 77), the latter indicating that this method may even be preferable when conducting interviews on sensitive topics (78). The alternative to conduct the interviews over phone was chosen by five of the interviewees, mainly because it allowed for greater flexibility in scheduling the interview at a time that suited them. The interviews lasted between 35 min and 1 h 45 min (average duration = 47 min). Each interview was recorded in its entirety and transcribed verbatim. Participants’ personal details were removed from the transcripts to preserve anonymity and the recordings were deleted once the interviews were transcribed and checked. All participants received a copy of the transcripts after the interviews along with the information that the recording had been deleted.

### Participants

Seven peer supporters and KRIS employees (five women, two men) in three local associations that work actively with different gender equality projects were interviewed for this study. Four participants have eight or more years of experience in KRIS, three have between 4 and 6 years of experience. All participants have lived experience of incarceration, criminalization, and substance abuse. The mean age of the participants is 46.7 years. To increase anonymity, their individual age or local association will not be disclosed.

### Data analysis

The interview data were analyzed thematically with the attempt to describe participants’ perceptions and experiences of the organizational changes and developments regarding equality during their employment/commitment in KRIS. The coding was made using the NVivo software. For this study, the interviewees were explicitly asked to account for organizational developments in relation to internal conflicts and the #Metoo-movement. I view the interview data as organizational narratives, defined by Vaara et al. [(79), p. 496] as “temporal, discursive constructions that provide a means for individual, social and organizational sensemaking and sensegiving.” Narratives of organizational change shape understandings of things that have happened in the past, as well as trajectories of the future (80, 81). The interviews touched upon several themes that related to (in)equality and organizational development, for example specific events/conflicts related to gender inequalities and victimization, descriptions of organizational changes, then versus now-narratives, equality work/projects, masculinity and power, and strategies for change. I themed the interview data based on how the problem with inequality in the organization was framed, how the organizational changes were explained, and what solutions were suggested. Change almost always involves narrative representation because of its immanent temporal development (79). The participants often talked about developments in KRIS in terms of “then versus now.” This narrative involved the phrase “flipping the triangle,” which was used to describe how the organization has gone from being run top-down to bottoms-up. I interpreted this as both an organizing narrative (in that changes for equality was centered around it), and a narrative that shaped the organization (KRIS was dysfunctional *before* but is *now* more democratic and well-functioning).

In the study materials I specifically looked for practical examples of the *how’s* of the organizations’ equality work as a supplement to the interview material.

## Findings

### Framing the problem

On one level, the interview narratives tie the problems and harms regarding inequality and sexism within KRIS to certain individuals. IP3 (F) says that the core values of KRIS (honesty, abstinence, solidarity, and comradeship^10^) really appealed to her when she first came to the organization, but that “the primary goal of KRIS was being lost because of specific individuals” who made KRIS an unsafe and dysfunctional space. These individuals are described as having caused harm to fellow members in the organization as well as to the organization itself. Central in this narrative is the image of harm as a “sickness” that affected the whole body of the organization. IP1 (F) similarly says that:

Many local associations have been ruled from the top. Some have lined their pockets in various ways and all of that. And maybe not everyone would dare to say, “this is what it’s like in my local association,” because then you’d get shit for it. You see what I mean? So, it’s good that we’ve cleaned out this sickness (IP1, F).

Below, the narrative of IP7 (M) interestingly problematizes the consequences of certain lived experiences of “the criminal life” on how the organization has been governed. Framing the “criminal attitude” as permeated by “power, control, and domination techniques” IP7 paints a picture of an organization where members have been afraid to speak up for fear of retaliation:

The board of RIKSKRIS has a responsibility and the chairman of RIKSKRIS only has one task, and that is to lead the board. It’s the board that makes decisions. And I actually think that throughout the years that KRIS has existed, people haven’t understood this concept. And that’s what’s frightening, because you’ve kept everything that you had with you when you came to KRIS, you see? From like the criminal life … and of course it was that attitude and that history that was there in KRIS that we like needed to bring up to the surface and start to change. So, it was like that those who’d been there the whole way just had to take a good look at themselves and see that, shit, yes … we’ve sat here like puppets. We haven’t dared to assert ourselves. We haven’t dared to speak up when we think that things have been done in the wrong way, because there has always been a leader or a chairman who is bloody loud and like, a lot of power, control, and domination techniques. And then it’s like … yes, but there’s been a sickness at KRIS for a very long time. (IP7, M)

Group secrecy and silence has been described as effective ways of legitimizing and maintaining abusive behaviors (82), something that is also highlighted in the quote above. On a structural and organizational level, the participants describe KRIS as a historically patriarchal organization with “the same hard jargon as it was out there [in the criminal lifestyle]” (IP2, F). IP7 (M) says:

There’s no need to make any pretense about the fact that KRIS has been a male-dominated association and organization ever since it started. You only have to look at the composition of the boards and the chairmen at the local level. So, women haven’t wanted to come to KRIS. And then you must ask: why is that? And it’s precisely this macho culture that’s existed, the biggest, strongest, loudest – that’s the one who decides things. And then I’d have to say that you haven’t changed the criminal mindset. You may have moved to a different playground, but you’ve kept all the criminal attributes. (IP7, M)

IP4 (F) says that when she first came to KRIS, the whole organization was male dominated: “The board, all leading positions, were held by men. Many of us women didn’t even get a chance to make our voices heard.” Peer support has been described as a liminal occupation (71), the peer supporters operating from the position of being both inside and outside the experience of the criminal justice system. IP4’s statement suggests that female peer supporters occupy a doubly liminal position, being viewed as outsiders within the PESO where their experiences of the criminal justice system have not been equally valued. IP4 continues:

I think that many have been seriously manipulated, I mean there was a hierarchy that was like set in stone. Among the men, I mean. And even if there were men who were not involved in this, I think the majority have been seriously manipulated by like an attitude. That’s what was difficult, which meant that like, yes … something was needed to break it. I mean, what was needed was for the chairman of the association to leave, and for people to like really put their foot down. And it’s not just about this chairman, but rather it’s about standing up for, like standing up against a behavior and a prison jargon that’s been like very firmly established in KRIS. (IP4, F)

In the interviews, this jargon is described as “macho,” “sexist” and “vulgar.” According to the participants, this kind of jargon has particularly severe consequences for women. IP7 (M) says that “we know from our own experience that women involved in substance abuse are exposed to a great deal,” and IP1 (F) emphasize that for women, the experiences of sexual abuse and violence is often intimately connected to their substance use, “that have been like a kind of salvation.” Along the similar lines, IP3 (F) say:

It shouldn’t be okay that like men who’ve been a long time [in the organization] go and grab the arses of girls who are new. Everyone should feel safe here. Whatever KRIS-organization you go to, I mean women in our target group have lived with extreme vulnerability, and presumably that’s the main objective: to come to a KRIS association wherever you might be, and that you will feel safe there. (IP3, F).

The perception that the support organization should be a safe space is repeated in the interviews. Against the backdrop of the particular vulnerability that is described here, being subjected to a peer support environment where patterns of sexualization and abuse continues, risk retraumatizing women and even affect their efforts to uphold desistance [cf. (35)]. IP3 (F) touches upon the personal consequences that being in this environment had for her:

When you come from this world [the criminal lifestyle] where I come from, this [sexism/sexualized talk] is normal. And like, you’re so used to it that you just kind of just let it happen. And when you’ve lived like this, with crime and substance abuse, and it’s something that carries on… I saw this as something common to many of the men in our organization, the leaders and among others in senior positions in KRIS, leading figures and so on, this like sexism and that. But then I have to say that you get colored by this yourself too. So, I’ve also had periods when I’ve been very unwell. I mean, I became ill after a few years of this; I ended up in a process of relapse. I’m not blaming it [the relapse] on the people at KRIS, absolutely not; I have a responsibility myself of course. But I stopped taking care of myself, and I became very affected by this. (IP3, F)

The narrative implies that the sexist environment had a strong negative impact on her sense of self-worth. She says she felt disheartened, assuming that “this must be what it is like everywhere then.” During her period of relapse into drugs, she says she “lost it completely,” ended up in a very destructive relationship and had to fight hard to reconnect with herself and to “find my way back to this good person that I am, and my own values and all that.”

Taken together, the framing of the problem with sexism and sexual harassment in KRIS that these narratives present has several similarities with the different framings of the problem that were constructed in Swedish #MeToo petitions [see (41)]. Firstly, the problem in KRIS is understood as connected to male-dominance and macho culture, where certain bullying behaviors and sexist jargon are seen as inherent. Secondly, sexism and inequality are constructed as residues of power structures from the criminal lifestyle, in which women are described as particularly vulnerable. Thirdly, the KRIS representatives mention collective behaviors that they believe have enabled the problem to continue, such as a culture of silence and processes of normalization. Similar frameworks, which situates the problem as systematic and structural, was evident in the Swedish #MeToo-movement (41). However, the peer supporters also make connections to specific aspects of their lived experiences of criminality, drug abuse, and (re)integration. This includes references to a “criminal mindset” that some men are still alleged to possess, and a particular vulnerability with regards to violence in their female target group. These gendered structures are seen as shaping and reproducing victimization in the space of peer support.

### Framing change and changing practice

The following section will explore the representatives’ framing of change within the organization, along with an analysis of practical changes and changed practice. Several participants mention that that there have been driving spirits in the organization before who have fought to improve the conditions for women, but that they have met strong internal resistance from men “at the top.” IP6 (M), for example, says that “the resistance has been bubbling under the surface. We have wanted to let women in, but it’s the ones at the top who have not wanted to have the women involved in that way.” Although the framing of sexual harassment within the organization was similar to models of explanations that were present in the #MeToo discourse (as shown above), most participants did not spontaneously link the organizational changes in KRIS to the #MeToo-movement. However, when asked, they made connections between this broader societal event and the course of development in KRIS, as the example below shows:

IP3 (F): When I came back to KRIS after my relapse treatment, we started working extremely hard to bring about change. And I and [colleague] became sort of spokespersons, like, “No, but now this has to change!” So, we started talking about everything that had happened, from the time I came to KRIS and this manager [name], who said that “she could do with a good licking out,” like totally sick stuff … that we ourselves had normalized. But I mean, things started to change years before that time, but this was like when it *really* started to change.

INTERVIEWER: So, what was different this time then?

IP3: There really needed to be an uproar. That’s what was needed, and more strong women could like bear witness to this.

INTERVIEWER: And was there a link to #MeToo? There was #withoutasafetynet and like other campaigns that happened at around the same time.

IP3: Yes, I mean, I think there is, certainly, because of course women in general have like started to talk more. And then it’s normal that it happens in KRIS too. I was also involved in #withouthasafetynet from the beginning, so I probably took a lot of that with me as well, to KRIS. We also had a lot of stories sent to us during this crisis with [individual in a senior position]. There were more women who like came forward who had been victimized by him.

Several others also link changes in KRIS to #MeToo as an overarching discourse that led women to start sharing their stories. IP1 (F) states that “[after #MeToo] then people dared … people dared to say this was what it was like; you know,” but women’s improved conditions in KRIS are also understood as dependent on changes that took place at the highest level of the organization, as evident in the conversation between IP4 and IP5 below:

IP4: I definitely think that #MeToo played a part [in the changes in KRIS]. But it wasn’t like we women decided to like “*now* we’re going to rise up.” It really just happened, you know.

IP5: The women in KRIS maybe like found the courage to lift this problem, that someone started talking about it because we found strength in this #MeToo-talk. So, it was a bit like “have you also been exposed to this?” “Yes, we have,” and then it got going quite quickly, and then you have to do something about it. Previously, a lid has just been kept on it all.

IP4: Yes, but I think it was significant that the change also took place in RIKSKRIS, because when it changed up there, we [women] were suddenly allowed in and people started listening.

A central theme in the narratives of change is the sexual harassment accusations against the former president of KRIS as *a turning point for the organization*, not only in terms of gender equality but in terms of democratic governance. This narrative is also the official organizational narrative that was published in KRIS magazine Vägen Ut [(74), issue 3]. The democratic decision to add *Equality* as a fifth organizational maxim [alongside *Sobriety, Honesty, Comradeship, Solidarity*] in 2021 was aimed at the core of the organizational identity and symbolizes the development toward implementing a feminist analysis that rejects the idea that gender equality would follow naturally upon an ideology of comradeship and solidarity. IP4 (F) says that “there was actually a group of men [in KRIS] who thought, no, but come on, we’ve got these maxims and that already includes gender equality!” The same argument against introducing equality as a fifth maxim is recalled by IP3 (F):

Before, the discussions were a bit, “But why should we have a maxim on gender equality? That should be covered by *Solidarity* and *Comradeship*.” And so we women said, “But it obviously isn’t.” And if we have a maxim to point to, like gender equality, then that’s that! If you don’t abide by it, then you can’t be part of KRIS. Then you can get excluded, and then there are consequences for not abiding by it. I mean we have consequences if you don’t abide by staying drug-free or behaving honestly. I mean, I lost my job and like, you know. And we think that gender equality is just as important. And to get that kind of resonance in it, we needed to make it a maxim in its own right. (IP3, F)

These discussions revealed the organization’s marginalization of women’s lived experiences both outside and inside of the organization and made the limits of comradeship and solidarity visible (like in many other social movements). In the wake of the accusations of sexual harassment, the chairman of KRIS was excluded from the organization. This event can be connected the to the raised concern that “[e]ven as a house cleaning is necessary, many worry that #MeToo’s victories will be short-lived in the absence of deeper structural and cultural changes” [(58), p. 54]. The question then is whether KRIS have done more than a house cleaning that could render more sustainable organizational changes. In the interviews, participants describe how the organization itself has had to undergo changes, not only regarding the male dominance and idealizations of hyper masculinity, but in terms of increasing democratic management. For example, IP7 (M) says that:

All members should be able to come and there should be no hierarchical order or any feeling of ‘oh, he’s the one in charge’. It’s not one individual who decides things, there’s a board, there’s an associational structure that has a democratic foundation.”

The question of responsibility is addressed in the interviews (and in the magazine), along with accounts of how KRIS have handled the problems and harms, and how they work to prevent such harms in the future. One change that has been made with regards to strengthening the democratic foundation of the organization is the introduction of the national two-day KRIS conference: *the KRIS-days*,^12^ hosted quarterly. Some local associations take part in the weekly #Kvinnostrejk, a women’s strike inspired by similar strikes in other countries with the aim to end systematic discrimination and oppression of women (83). IP1 (F) emphasizes the fact that “the guys [from KRIS] are also there [at the strike]. We think that that’s important, because this is a man’s issue to a great extent and men usually listen more to other men.” At the same time, she says the women sometimes struggle to get men with experiences of criminalization and substance abuse to really understand women’s experiences:

IP1 (F): The men just don’t have the same experience as we women of what it’s like in the active [criminal lifestyle]. And that makes it difficult to understand some aspects. I’m not saying that they must understand it straight away, but it’s difficult for them to relate to some of the things that you’ve been subjected to. A lot of them think like, “Yes, but what are you talking about, why didn’t you leave him?” It’s a bit of an uphill struggle, you know. […] If you’re a woman who’s been exposed to violence, then getting hit left and right is normal, so you have to start with this, as I say, broad understanding. This is what it’s been like, can you all understand that? And then you have to refine it to, “Yes, can you understand what it’s like when you’re trying to become part of a KRIS association?”

One of the changes of practice within KRIS that aim to increase knowledge and identification is the introduction of the *study circle* “The meaning of violence” where the members meet weekly to discuss violence related themes. For example, the theme of sexual violence includes questions like: *What is sexual violence? Have I been subjected to any kind of sexual violence? Have I subjected others to any type of sexual violence? What are my thoughts on sexism and macho culture? How can I as an individual take responsibility for a more equal society and counteract sexism and sexual violence? How can we as an organization take our responsibility to counter sexism and sexual violence?* The discussion themes in the study circle are interesting from a strengths-based perspective and build upon both generative, feminist, and restorative approaches. The questions concern lived experiences of being both victim and perpetrator of sexual violence, and highlights members’ own values and attitudes. The questions also take a wider perspective, challenging the participants to imagine themselves contributing to a more equal society, as well as taking part in building and developing a PESO with a feminist mission.

IP1 (F): We have a fantastic study circle where we go through all the forms of violence that exist. What *is* violence? And I think I know a lot from my own life, but I’ve learned an awful lot. […] You get to learn about material violence, for example, that a lot of people think isn’t such a big deal. I haven’t lived in a single apartment where there hasn’t been a hole in every wardrobe door! Or like destroying somebody’s things. “Yes, but I didn’t hit you, I just destroyed your clothes” … or your mobile phone or whatever it might be. And this latent violence, that people don’t talk about very much. What it can be like in families where it just sits there like a dark cloud over… like when is dad going to smash his fist down on the table? You hardly dare to breathe and can hardly eat. […] We also talk a lot about sexual abuse, which is a very taboo subject. We women are a bit more in the forefront and dare to talk about it. But you know that many men who’ve spent time in prison have been exposed. So, it was really good at the KRIS-days, when there was a guy who opened up about it. And how we then need to like … because it’s not *your* shame that you have to carry! (IP1, F)

The interviews as well as the study material show that KRIS also extend victimization to include men as victims of patriarchal norms and violence, but also as ultimately responsible for ending violence and changing norms.

IP3 (F): With women, we work with what they’ve been subjected to, a lot of sexual violence and that kind of thing. With the men, we work of course with what they’ve subjected others to. And then you have to strike a balance, so that the women and the men can cope with being here together. And that the men can like talk about, were they allowed to be sad when they were children? And what does it mean being a man in like a criminal environment … so that’s probably the big differences, that among the men we have quite a lot of perpetrators in one way or another. I’m not saying there’s a load of rapists, but in one way or another [they are perpetrators]. And a lot of guys that come here don’t understand either… but “oh, is that violence? Many men don’t understand that they’ve subjected women or girls to violence, so we like work to raise awareness about that, and to start talking about it. Because we think it’s the men who can have a major influence. Influencing and teaching other men. So that’s a big responsibility, that we start with them.

The feminist development and the analysis of masculinity and male responsibility affect KRIS’ strengths-based practices in several ways; new projects have formed with the intention to support and educate male peer mentors that can pay the message forward [cf. (84)]. Raising male awareness of violence, sexism, victimization, and responsibility is one of the main themes that are addressed in KRIS men’s groups where different questions are discussed, including: *what is important to me in an intimate relationship? Was I allowed to be sad when I was a child? Am I complicit in the macho culture? How do I react if a friend makes a sexist joke? In what way has my upbringing influenced my values around gender norms?* In the men’s group, the discussion questions focus on lived experiences that go beyond direct lived experiences of substance abuse and criminality (such as sexist jargon and suppressed emotions), but which, from a feminist perspective are clearly linked to masculinity, power and ultimately violence.

## Discussion and conclusion

This article analyzed feminist organizing in a Swedish PESO for people with experiences of criminalization and substance abuse in the aftermath of #MeToo. The study shows that there are several parallels between how the issue of sexual harassment was framed by KRIS and the way this issue was framed by the #MeToo petitions that emanated from other Swedish workplaces and industries. For example, the outline of the problem as a democratic issue rather than a women’s issue and a question of providing a safe place for all members (i.e., a work environment issue), is aligned with the problem definition of the broader #MeToo-movement in Sweden (41, 56). The interviews also indicate a raising of awareness among women in KRIS that came from sharing experiences of sexual harassment and realizing they were not alone. Similar to the effects that #MeToo had in terms of revealing organizational problems such as male cultural power, ostracism, and different kinds of misconduct (53, 55), the internal accusations of sexual harassment in KRIS uncovered deeper issues of power conflicts and a culture of silence.

A number of measures have been taken in the organization in order to give voice to women whose livelihoods are affected by crime, imprisonment, violence, and drug abuse. These measures include a campaign for sexual consent, a podcast about violence toward women and children, and workshops that critically discuss masculinity and violence. The study indicates that the #metoo-movement facilitated changes that were already starting to take place within the KRIS organization, and that these broader societal events helped mobilize women within KRIS and further legitimized their demands to deal with the organization’s macho culture. The framing of sexism and macho culture as part of a “criminal mindset” challenge micro- and meso-level relational desistance in the peer support context as it questions whether a person upholding these attitudes have really changed. While this may put individuals in a liminal state of being neither offender nor recognized as someone who have “made good” (19), it also expands KRIS’ definition of what it means to desist from crime and ultimately changes how the organization works to support identity desistance. By extending their strengths-based peer support activities to also include the collective challenging of masculinity norms as well as arranging men’s groups that involve exploring vulnerability, emotional abuse, and responsibility, KRIS is imagining what feminist, restorative (re)integration could look like. The study thus uncovers how power structures can be challenged by putting the gendered lived experiences of women with a history of criminalization and substance abuse in the center of ex-offender peer support.

If the #MeToo-movement has taught us one thing, it is that there is absolutely nothing unique with an organization or workplace environment where women are sexually harassed. In that regard, what happened in KRIS was just business as usual. What sets KRIS and similar PESOs apart from most other organizations or workplaces is the stigma that is attached to them as organizations run by people with criminal records and previous addictions. Researchers in Social Work as well as in Criminology have pointed to the “liminality inherent in peer support” [(71), p. 188], where “little [is] needed to topple perceptions of ‘progress/rehabilitation”’ [(85), p. 10]. PESOs occupy an organizationally liminal position, always located in the focal eye of risk management. Actual or suspected misconducts such as in The Swedish Inheritance Fund incident (86) risk tarnishing the entire organization’s reputation, leading to the loss of funding or canceled contracts with other criminal justice actors such as the Prison and Probation Service, which jeopardizes the organization’s support services. The accusations of sexual harassment also gave the organization negative media attention and caused internal conflicts. Against the backdrop of liminality I suggest that this study’s narratives framing of problems and solutions in KRIS can be thought of as “restorative storytelling that redefines an ethical conception” [(87), p. 10] of the organization, functioning as a shame management tool for resisting stigma attached to the harms committed by its liminal subjects.

The liminality of peer support is further complicated when considering the gendered power structures described by the interviewees in this study. Female peer supporters with lived experiences of criminalization and substance abuse occupy a doubly liminal position; not only are they both inside and outside the experience of the criminal justice system as peer workers [cf. (71)], but they are also in a state of being in-between sexual objects and peers, as outsiders within the PESO where their experiences of the criminal justice system have not been equally valued. As research shows that peer supporters run the risk of being retraumatized while using their lived experience in the practice of helping others (7, 85), this study’s uncovering of women’s lived experiences of trauma within peer support practices and their struggles to redefine the foundations of their organization adds to the understanding of lived gendered emotionality of peer support. To conclude, the focus on feminist organizing in peer support narratives and practice also says something about *belonging* in the sense of “*being a recipient of social goods* (that is, someone enjoying fair access to all the resources, rights and opportunities routinely afforded to other citizens)” [(20), p. 436]. There are clearly gendered barriers to belonging and to becoming a recipient of social goods, as the resistance to unequal support for desistance in this study shows.

### Limitations

This study is based on the developments in one organization as told by a limited number of informants. Although the interviewees have long experience of working in KRIS and can be defined as representatives of the organization, there are probably other stories of the changes in KRIS that this study does not reach. Furthermore, since the organization’s work for increased equality and raising awareness of destructive masculinity and violence is in its early stages, some of the narratives speak more of changed values than of practical changes. Additionally, the “new” organization is still in the making and its future is not clear. Whether or not KRIS manages to implement their new maxim in all the local associations, and what effect the organizational changes might have on membership numbers, reputation, funding, and collaborations with other criminal justice actors is a question for follow-up studies.

## Data availability statement

The original dataset presented in this article are not readily available because they contain potentially identifying or sensitive personal information. Inquiries about the primary data can be directed to the corresponding author.

## Ethics statement

The studies involving human participants were reviewed and approved by the Swedish Ethical Review Authority (reference number 2021-05339-02). The patients/participants provided their written informed consent to participate in this study. Written informed consent was obtained from the individual(s) for the publication of any potentially identifiable images or data included in this article.

## Author contributions

The author confirms sole responsibility for the following: study conception and design, data collection, analysis and interpretation of results, and manuscript preparation.

## Footnotes

1 Convict Criminology have been accused of excluding marginalized groups such as women, ethnic minorities and LGBTQs (90). Representatives acknowledge that white men are overrepresented in the field (22), but emphasize that multiply marginalized individuals may have good reason for not coming out as ex-convicts, and that the critique reveals a lack of insight into the effects of intersecting stigmas (91).

2 Safe spaces are commonly discussed and practiced in feminist, queer, and civil rights movements as spaces where marginalized groups are shielded from violence and harassment (92).

3 #Withoutsafetynet started as a #MeToo-petition in 2017 and became a non-profit organization in 2018. The organization also includes transgender people.

4 #Notyourwhore likewise started as a #MeToo-petition in 2017 and transformed into a non-profit organization in 2018. The organization’s target groups are women, non-binary, and transgender people.

5 Acronym for Criminals’ Return Into Society (in Swedish: Kriminellas Revansch I Samhället).

6 The organization has also been active in Finland since 2003 (www.kris.a.se; www.kris.fi).

7 Young KRIS started 2006 and target young people between the ages of 13 and 25 (www.ungakris.se).

8 An annual political festival in Gotland, Sweden.

9 In 2022 KRIS was granted funding for making prison visits in Stockholm and Gävle (https://www.kriminalvarden.se/om-kriminalvarden/samverkan-och-samarbete/statsbidrag-ansok/organisationer-som-fatt-bidrag/).

10 The fifth maxim “Equality” was added in 2021.

12 The first KRIS-days conference was held in September 2020.
